# Office Visitors

**Published:** 1907-02

**Authors:** 


### OFFICE VISITORS

He appeared to us a man of between forty and fifty years of age naturally, but unnaturally as old as one between sixty and seventy. Wrapped in a heavy overcoat he shivered in a temperate atmosphere. An examination of his mouth caused us to recoil. He noticed it and said, "I do not wonder at your surprise, Doctor, for I am in a fearful condition. I have led the pace that kills and from a physically perfect and well-educated man, with wealth, to back me in all my endeavors, I am a broken down remains of a fast life, in which 'wine, women and song' have played the most important parts. I am under medical treatment for a constitutional disorder and my physician has recommended that as a necessity I have my mouth put in order. I now have little means, so be as liberal with me as possible in your charges. You are a stranger to me professionally and that is the reason I apply to you. I am ashamed to present myself to my former dental friends for various reasons."

His mouth was a wreck in consonance with his body. The latter permeated with the germs of that fearful disease that is the curse of such a life. The mucous membrane was patched with ulcers. Pyorrhea and exposed nerves abounded. In such a plight, had he not a vigorous natural constitution his life would have ended speedily, but it held together wonderfully. We made a treatment and with an appointment dismissed him. Of course prudence caused us to make inquiry of him and we found the tradesmen would refuse to credit him and professional friends opined that poverty and pride kept him away from them and that he would rather "do" a stranger than old friends. After deliberate thought, compassion for the poor fellow got the best of us and we fixed him up in good shape and made an ample deduction from the bill, though his case required the utmost care and diligence in operating to protect ourself and others from the virus of his diseased mouth. To our surprise and delight he was very grateful and appreciative and paid the bill, with the remark that if he "was in the condition of former years" he "would double the amount gladly," for he knew that "we understood his case and respected his feelings and regarded his financial plight." He wished for us every good thing in life and that as he had enough left to get to the Hot Springs, if he got well, on his return he would ever after be our fast friend and patient.

